# Extending Lawson and Robins’ (2021) guideline for the evaluation of jingle and jangle fallacies

**DOI:** 10.3758/s13428-025-02691-6

**Published:** 2025-05-19

**Authors:** Christian Blötner

**Affiliations:** Faculty of Psychology, Chair of Personality, Legal Psychology and Assessment, Universitätsstr. 37, 58084 Hagen, Germany

**Keywords:** Validity, Dependent correlations, Construct overlaps, Assessment

## Abstract

The existence of *jingle fallacies* (equally named constructs/measures that, in fact, assess different constructs) and *jangle fallacies* (differently named constructs/measures that, in fact, measure the same concept) jeopardizes psychological assessment, as both are associated with conceptual and assessment-related uncertainties. A guideline presented by Lawson and Robins *Personality and Social Psychology Review*, *25*, 344–366, (2021) helps evaluate the intensity of respective fallacies. While the guideline is well elaborated, psychometric aspects regarding (dis)similarities of nomological networks require extensions and differentiations. I recommend two analytical advancements, namely (a) the derivation of correlation difference hypotheses for criteria with which the allegedly jingled (jangled) variables are assumed to be correlated at equal (different) levels and (b) procedures to derive cutoffs for the overall similarity of nomological networks based on the *elemental approach* (Kay & Arrow *Social and Personality Psychology Compass*, *16*, e12662, 2022). Considering correlation difference tests, I further outline the importance of power analyses. These extensions help improve the evaluation of assumed jingle and jangle fallacies, arguably increasing the stability and reliability of research findings.

Psychology differs from other scientific branches in terms of the visibility of its subject matter. One day, technical advantages may enable physicists and chemists to make even subatomic particles visible, but psychologists will never directly observe psychological characteristics. For this reason, it is not easy to conclude at first glance whether the intended psychological constructs at hand are measured accurately or whether the employed instruments rather assess closely related yet distinct constructs. Consequently, research psychologists frequently postulate ostensibly new constructs that turn out to be redundant with readily known constructs only after at least a few studies have been conducted on the allegedly new construct. Likewise, different researchers often come up with different definitions for the same constructs, leading to different conceptualizations and, thus, non-convergence of the measures. Erroneously treating concepts/measures as distinct because they have different names is called a *jangle fallacy*; falsely claiming that concepts/measures are identical because they are named equally is called a *jingle fallacy*. Both pose serious problems because they exacerbate the comparability of findings due to heterogeneity in assessment and account for the instability of findings and waste of research resources (Hodson, 2021).

Owing to the above problems, it is important to be able to identify jingle and jangle fallacies at an early stage, ideally in the derivation study for a new construct/measure. Lawson and Robins (2021) provided a respective guideline with 10 criteria that can be evaluated independently so that different conclusions can arise from different criteria. Thus, jingle and jangle fallacies differ on a spectrum rather than being dichotomous conditions, suggesting that it is more appropriate to talk about weak, moderate, or strong evidence for or against a jingle/jangle fallacy than about presence versus absence. To acknowledge this, they introduced the term *sibling constructs* to refer to constructs that “share a close, familial relation, but are not identical; that is, they are not ‘twin constructs’” (p. 345). It stands to reason, however, that perfect twins rarely exist in the realm of psychological constructs. Following the principle of theoretical parsimony, researchers frequently treat constructs as equal if there is little conceptual and empirical space for uniqueness. Thus, postulating a “new” construct only pays off if it reflects a sibling construct in the sense of Lawson and Robins but not a twin construct in the sense of a jangle fallacy. In this regard, Lawson and Robins (2021) themselves consistently used the jingle/jangle terminology in their paper and applied their framework to examples of (assumed) jingle and jangle fallacies (not just what they called sibling constructs), making the framework universal to use.

Specifically, Lawson and Robins’ (2021) framework evaluates conceptual similarities of definitions (1), similarities of the *hypothesized* and *empirically observed* nomological networks (2 and 3), correlations of the measures (4), the constitution of common vis-à-vis separate factors of pertinent measures in factor analyses (5), mutual incremental validity for crucial criteria (6), the existence of shared developmental routes, a common cause, or even a causal relationship (7 to 9), and whether the ostensibly jingled/jangled concepts are state or trait manifestations of the same entity (10). Problematically, it remains unclear where to draw the line to decide, for a given criterion from the framework, whether or not two constructs are sibling constructs. The same problem applies to decisions about jingle and jangle fallacies, however, requiring researchers to provide more detailed considerations about the potentially subjective bases of their decisions—regardless of whether they rely on the sibling or the jingle/jangle terminology. The current manuscript describes psychometric refinements of Lawson and Robins’ (2021) framework that can be used for evaluations of jingle/jangle fallacies and sibling constructs alike.

The guideline itself is valuable as it provides a structure of criteria with which many scholars expectedly agree. However, it stands to reason that the recommended approach to evaluating distinct *expected* nomological networks (Criterion 2) oversimplifies conclusions about jingle/jangle fallacies and that Lawson and Robins’ (2021) proposed procedure to evaluate the equivalence of nomological networks (Criterion 3) relies on unrealistic premises. In this research, I propose extensions of these aspects that call for stronger incorporation of conceptual descriptions of the evaluated constructs (cf. Criterion 1) and empirical overlaps (Criterion 3). Stronger emphasis on these aspects arguably helps quantify the extent to which a jingle/jangle fallacy (or sibling constructs) exists and advances the transparency of respective evaluations.

## Expanding on “high degree of overlap in their actual nomological network”

### Directional hypotheses versus difference hypotheses

The guideline by Lawson and Robins (2021) remained comparatively vague about how to analyze the nomological networks of potentially jingled/jangled variables. In fact, there are different ways to derive hypotheses for expected nomological networks, but they differ concerning their specificity. The easiest way refers to postulating the direction for each correlation: Let *X* and *Y* be constructs for which a jangle fallacy is to be tested, and let *A* be a validation criterion with which *X* and *Y* should be correlated. A phrasing for directional hypotheses would be “*X* is expected to be positively correlated with *A*, whereas *Y* is expected to be negatively correlated with *A*.” This type of hypothesis is informative if correlations are assumed in different directions, but it is associated with a massive loss of information when it comes to evaluations of jingle and jangle fallacies if positive/negative correlations are assumed for both *X* and *Y*. A more informative yet more rarely adopted type of hypothesis refers to correlation differences. This type of hypothesis requires researchers to justify which of the competing constructs exhibits a stronger positive or negative correlation. An exemplary study employing the correlation difference approach dealt with the assumed jangle fallacy of *subclinical psychopathy* (hereafter psychopathy) and *everyday sadism* (hereafter sadism), both of which are characterized by aggression and dominance seeking (among others). Resorting to violence is focal to the definition of sadism, and dominance seeking is the assumed underlying motive. In comparison, aggression and dominance seeking are rather peripheral to psychopathy and reflect only two out of many features, leading the authors to hypothesize sadism to be more strongly related to aggression and dominance seeking (Blötner & Mokros, 2023). Similar correlations of measures of competing constructs with aggression, dominance seeking, and other important constructs suggested a jangle fallacy that would not have become clear in such detail through mere directional testing (for confirmations of these findings see Blötner et al., 2024; Kowalski et al., 2024). Lawson and Robins (2021) did not consider the potential for correlation differences, presumably leading most researchers to resort to mere directional analyses of correlations when they apply the framework. Arguably, it can be cumbersome to derive hypotheses about which construct/measure is most strongly related to an outcome or set of outcomes, but these considerations provide additional information about areas of overlap and reduce the chances of *falsely* claiming a jingle/jangle fallacy (see below).

### Overall agreement of nomological networks

Besides evaluations of bivariate correlations with crucial criteria, Lawson and Robins (2021) recommended computing the overall agreement of the observed nomological networks (spanned by a reasonable set of criteria) of the measures for which a jingle or jangle fallacy is tested. To this end, they suggested the *double-entry intraclass correlation* (ICC_DE_). To calculate it, the correlations observed between one construct/measure in question and the validation criteria are appended to the observed correlations of the other one and vice versa (see Table 1 for an illustration). The ICC_DE_ is the bivariate correlation between these doubly entered vectors of correlations. The double-entry method ensures that the resultant coefficient reflects the similarity of the coefficients rather than mere rank-order similarity.

**Table 1:** Illustration of the double-entry procedure of the double-entry intraclass correlation

	Correlations			Doubly entered correlations	
Criteria	*X*	*Y*		*X*	*Y*
*A*	*r*_XA_	*r*_YA_	**➔**	*r*_XA_	*r*_YA_
				*r*_YA_	*r*_XA_
*B*	*r*_XB_	*r*_YB_		*r*_XB_	*r*_YB_
				*r*_YB_	*r*_XB_
*C*	*r*_XC_	*r*_YC_		*r*_XC_	*r*_YC_
				*r*_YC_	*r*_XC_

*Note.* The first subscripted index refers to the assumed jingled/jangled construct/measure (*X* or *Y*), and the second subscripted index refers to the validation criterion (*A*, *B*, or *C*)

Lawson and Robins (2021) recommended ICC_DE_ ≥ .60 as a cutoff for equivalence. However, as they themselves noted, the agreement of nomological networks strongly depends on the selection of constructs constituting the nomological network. Furthermore, it stands to reason that empirical agreements of correlation profiles exceed this cutoff quite easily owing to common method effects, for instance, if all variables are measured with self-report scales or if all selected criteria, by and large, reflect very prosocial or very antisocial constructs, to name just two causes. In many cases, certain overlaps are inevitable because “everything correlates to some extent with everything else” (Meehl, 1990, p. 204). Previous studies that examined jangle fallacies applied a much higher cutoff than Lawson and Robins to treat nomological networks as equivalent (ICC_DE_ ≈ .90; Blötner & Mokros, 2023; Few et al., 2016; Hart et al., 2023; Maples-Keller et al., 2019; Miller et al., 2017; Samuel et al., 2012). Applying a fixed cutoff, however, neglects the complexity of shared and non-shared elements of nomological networks. Thus, the present study proposes an approach for the derivation of a cutoff that also takes content-related specifics into account.

Decisions in the context of evaluations of jingle/jangle fallacies can be compared to a diagnostic process in that a decision must be made about whether committing a type I or a type II error is more problematic: A type I error in this context would occur if researchers claimed a jangle fallacy where there is none (i.e., false positive), which could lead other scholars to treat the ostensibly jangled (yet factually distinguishable) constructs as equal. Imagine, for instance, that Blötner and Mokros (2023) committed a type I error in their study on the jangle fallacy affecting sadism and psychopathy because they adopted a cutoff for the overall agreement of nomological networks that was too low. Under these circumstances, the construct of everyday sadism could be erroneously eliminated from a research area even though it was, in fact, sufficiently unique vis-à-vis subclinical psychopathy (note that subsequent studies confirmed Blötner and Mokros’ findings [e.g., Blötner et al., 2024; Kowalski et al., 2024)]. A type II error, in turn, exists if researchers fail to recognize a jingle/jangle fallacy as such (i.e., false negative), for example, because they adopted a cutoff for the overall agreement of nomological networks that is too high. Both kinds of false decisions can be costly and detrimental to the underlying field of research: In the case of a type I error, useful work done by earlier researchers would be labeled as “useless” despite being, in fact, practically and conceptually useful. Thus, the financial, personal, and time-related resources invested in the researchers’ work would not pay off as intended. In the case of a type II error, the same kinds of resources would be “wasted” for upholding parallel strands of research for factually the same hypothetical construct (cf. Hodson, 2021). The decision as to whether committing a type I or a type II error is more costly should depend on the research area and the amount of work already devoted to the constructs under scrutiny. Thereby, unspecific cutoffs and requirements for the evaluations of the similarity of hypothesized and observed nomological networks (Criteria 2 and 3) can *erroneously* suggest a jingle/jangle fallacy or favor a respective oversight, especially if researchers do not acknowledge the conceptual proximity of the constructs (Criterion 1). Thus, Lawson and Robins (2021) recommended examining their 10 criteria simultaneously and integrating them for an overall judgment.

## Methodological considerations to advance Lawson and Robins’ framework

### Which numerical differences are treated as meaningful differences?

An important issue related to statistical evaluations regards drawing a reasonably large sample to test the underlying hypotheses of the study, because the sample size determines whether an effect size yields significance at a specified α-level. That said, benchmarks for the evaluation of effect sizes neglecting the concrete context appear useless, suggesting that researchers should justify which effect size they deem nontrivial for the concrete study (Giner-Sorolla et al., 2024; Riesthuis, 2024). In the context of jangle fallacies, researchers should provide strong reasoning about which numerical difference between correlation coefficients of two ostensibly jangled measures with any external construct they consider to be too small to be interpreted as a *meaningful* difference. Afterward, researchers should collect enough data to reliably detect this correlation difference, highlighting the role of a priori power analysis.

**Fig. 1:**
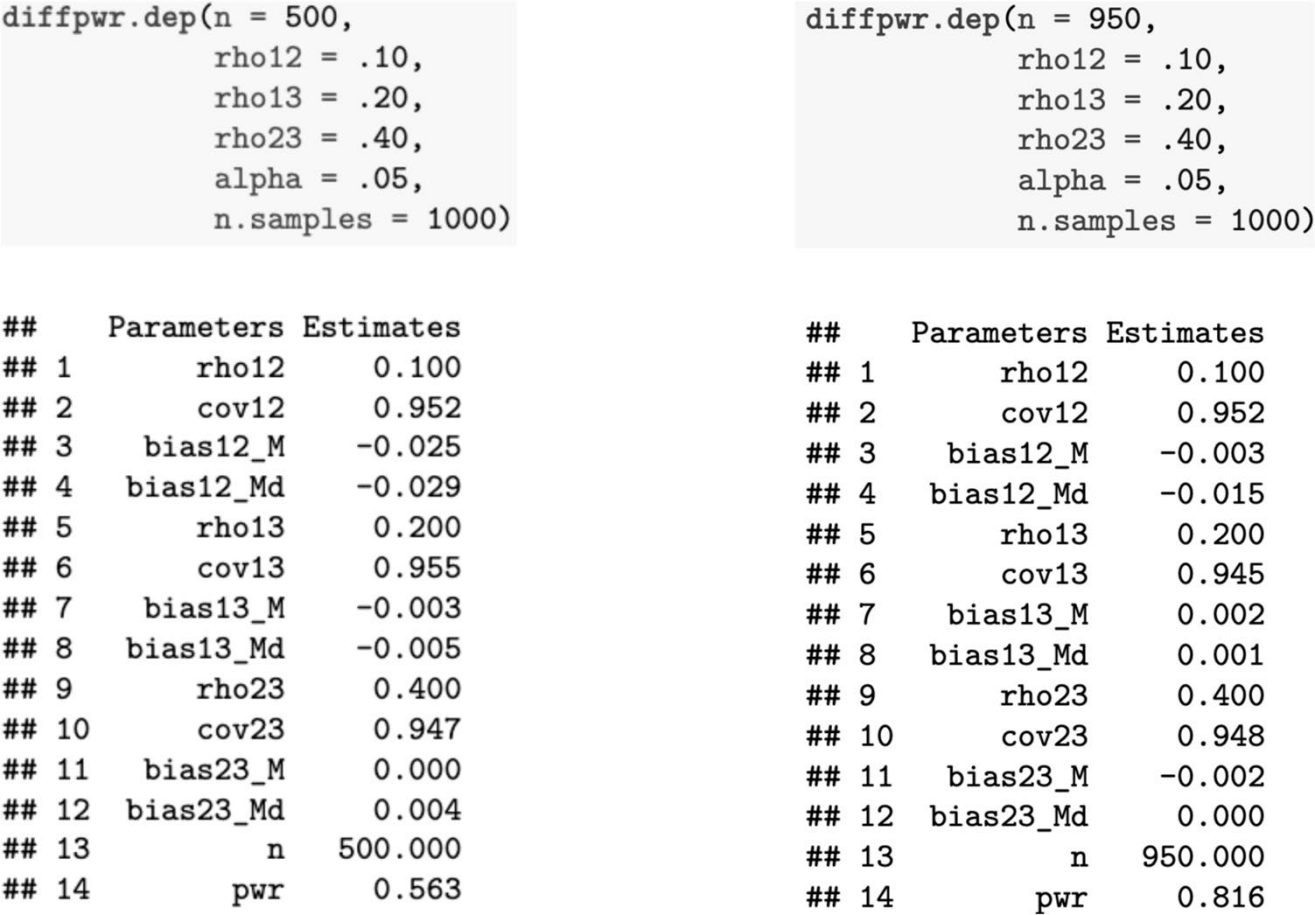
Illustration of the power analyses for the outlined exemplary correlation difference. *Note*. n = Tested sample size. rho12 = Correlations of the validation criterion with the first construct (equals *r*_XA_ from the text). rho13 = Correlations of the validation criterion with the second construct (equals *r*_YA_ from the text). rho23 = Correlations between ostensibly jangled constructs (equals *r*_XY_ from the text). alpha = Type I error level α. n.samples = Number of samples drawn for the simulation. Parameters denoted with cov in the output indicate the ratio of confidence intervals around the simulated correlations that include the target correlation. Parameters denoted with bias in the output indicate the relative differences between the mean (appended _Md) or the median (appended _Md) of the distribution of the simulated correlations and the target correlation. pwr = Achieved power 1−β. For more information, see Blötner (2024)

The R package *diffcor* (version 0.8.4; Blötner, 2024) provides Monte Carlo-based power analyses for correlation difference tests for dependent correlations (*diffpwr.dep*; see supplemental template *R* file under https://osf.io/v5dte/). Consider, for example, that a researcher decides to treat the difference between the correlations of the constructs *X* and *Y* with a validation criterion *A* Δ*r = r*_*XA*_ − *r*_*YA*_ ≥ .10 as meaningful and that they expect *X* and *Y* to be correlated with *A* at *r*_XA_ = .10 and *r*_YA_ = .20; at the same time, they assume *X* and *Y* to be correlated at *r*_XY_ = .40. Applying the template *R* script to this example reveals that a sample size of *n* = 500 is not sufficient to detect this correlation difference at a level that is usually deemed the lower bar for sufficient statistical power (i.e., 1 − β < .80; Giner-Sorolla et al., 2024), given an α-level of 5% (see left side in Fig. 1), but a sample size of *n* = 950 does (see right side of Fig. 1). In addition, the simulated data accurately reflect the target coefficients (indices denoted as bias and cov; see explanations in the *Note*).

Note that, depending on the intercorrelation of the ostensibly jingled/jangled constructs, this procedure requires larger sample sizes than approaches purported to ensure sufficient power to detect a bivariate correlation of prespecified size. Thus, a *correlation coefficient* as high as *r* = .10 yields significance at a smaller sample size than a *correlation difference* as high as Δ*r* = .10, given equal α-level and target power (Riesthuis, 2024). It may be appealing to reduce the necessary sample size by increasing the correlation difference deemed critical for (non)redundancy (e.g., Δ*r* = .20). Under these circumstances, ceteris paribus, 250 participants would be needed to detect the pattern outlined in the previous paragraph with a power of 83%. Note in this regard that a nonsignificant correlation difference due to a smaller sample size can lead scholars to falsely claim that two test scores are correlated with the validation criterion at similar levels although the descriptive difference (Δ*r*) is noteworthy. For example, Gignac and Szodorai’s (2016) effect size convention treats a correlation coefficient as high as *r* = ±.20 as a moderate effect. Therefore, I argue that robust and consequential research requires and deserves well-powered studies, suggesting that sound studies on jingle and jangle fallacies should not be too lenient by relying on way too small samples and/or way too large differences.

That said, power analyses per se cannot tell whether nomological networks differ, because, following the above reasoning on type I and II errors, statistically significant differences are not necessarily meaningful from a practical stance (i.e., sufficient power to detect even negligible differences), and practically meaningful differences do not always yield significance (i.e., insufficient power to detect meaningful effects). However, power analyses require researchers to reveal which (potentially subjective) criteria they apply to decide whether they treat (a difference between) effect sizes as meaningful or negligible (see Giner-Sorolla et al., 2024, for considerations about transparent reports and justifications of assumptions). For instance, if a study suffers from insufficient power to detect a target effect, differences between correlation coefficients that are noteworthy at a descriptive level could be erroneously treated as evidence in favor of a jangle fallacy because the statistical test does not yield significance at a specific α-level (e.g., *r*s = .10 and .30, given *n* = 100 and an intercorrelation *r* = .40, *p* = .06). If, in turn, the sample size of a study is sufficient to detect even negligible differences, researchers would oversee a jangle fallacy (e.g., *r*s = .10 and .12, given *n* = 4,000 and an intercorrelation *r* = .80, *p* = .04). Thus, the need to justify applied benchmarks when planning a jingle/jangle study renders power analyses a helpful tool to increase the transparency and reliability of respective conclusions.

### Context-specific thresholds for the agreement of nomological networks

I argue that considerations about a critical level of agreement of the nomological networks must take the *concrete* degree of differences and overlaps into account (see Criterion 1 from the guideline by Lawson & Robins, 2021). That is, if only small differences exist between two potentially jangled variables, the applied cutoff should be higher than that for a case of two variables that reveal at least a moderate degree of differences. If the index of the similarity of the observed nomological networks is smaller than the cutoff yet still high at the numerical level, a strong jangle fallacy can be ruled out, and the conclusion of sibling constructs as per Lawson and Robins would make sense.

As of yet, there is no consensus for an approach to derive thresholds for similarities of observed nomological networks that considers concrete commonalities and differences between the investigated variables. To address this, I propose a scheme that builds upon the integration of correlation difference hypotheses. This extension also serves a closer integration of Lawson and Robins’ (2021) Criteria 1 to 3 (i.e., conceptual differences as well as similarities of hypothesized and observed nomological networks): First, based on the underlying conceptualizations of the ostensibly jangled constructs, researchers need to select a reasonably large set of validation criteria for which distinct correlations are expected, but also criteria for which no differences are assumed (cf. Blötner & Mokros, 2023). Including criteria with which no differences are assumed ensures that shared features of both are also considered. Based on the comparison of the underlying theoretical frameworks, researchers can extract external constructs with which one of the ostensibly jangled constructs is expected to yield stronger relations.

**Fig. 2:**
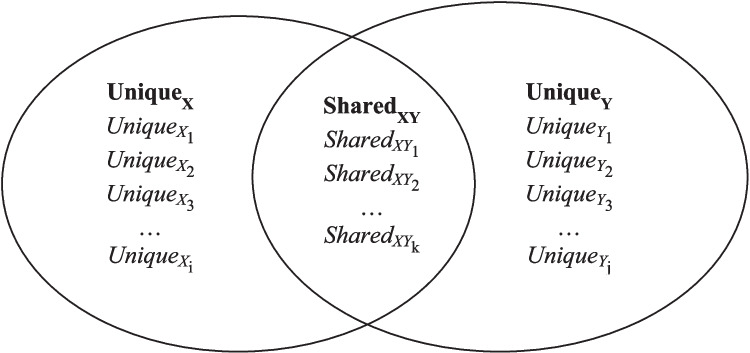
Schematic application of the elemental approach to derive unique and shared elements. *Note*. Unique_X_ and Unique_Y_ denote contents that are theoretically more important for *X* and *Y*, respectively. Shared_XY_ denotes contents that are (equally) important for both. The numbers of unique and shared contents (i, j, k) can but do not have to differ

To specify a set of shared and distinct contents, the *elemental approach* provides a helpful framework. It reflects the decomposition of theoretically important contents of the constructs under scrutiny and can be visualized by Venn diagrams (Kay & Arrow, 2022; see Fig. 2 for a schematic illustration). Based on the decomposition of theoretically important contents, researchers can derive operationalizations of classes of criteria. For instance, researchers may operationalize selfishness, which represents important content of psychopathy, through contributions to a *public goods game*.

After defining the central elements of the nomological networks, researchers can derive the threshold for (non)redundancy as the ratio of empirically detected correlation differences (denoted as *d*) and the total number of measured criteria (denoted as *t*). The critical threshold for the ICC_DE_ results from the formula ICC_DE, crit_ = 1 − *d*/*t*. For example, consider that a researcher administered 20 criterion measures (= *t*). In five cases, they detected significant correlation differences in the sense of the last section (= *d*). Applying the above formula, the cutoff for a jangle fallacy would be ICC_DE, crit_ = 1 − 5/20 = .75. If the empirically observed ICC_DE_ exceeds .75, the researchers would conclude that a jangle fallacy occurred. An adaptation of this procedure for assumed *negative overlaps* (i.e., one construct is assumed to reflect the opposite of the other [e.g., altruism and egotism]) requires an alternative counting approach for *d*: In this case, researchers count the number of correlation differences that differ in *direction* but not in (absolute) *strength of association* (i.e., |*r*_XA_| ≠ |*r*_YA_|). That is, empirically observed correlations *r*_XA_ and *r*_YA_ are treated as functionally different if *X* turns out to be more strongly *positively* related to *A* than *Y* is *negatively* related to *A* (e.g., *r*s = .30 and −.30 are functionally equivalent, but *r*s = −.20 and .50 are not).

**Fig. 3:**
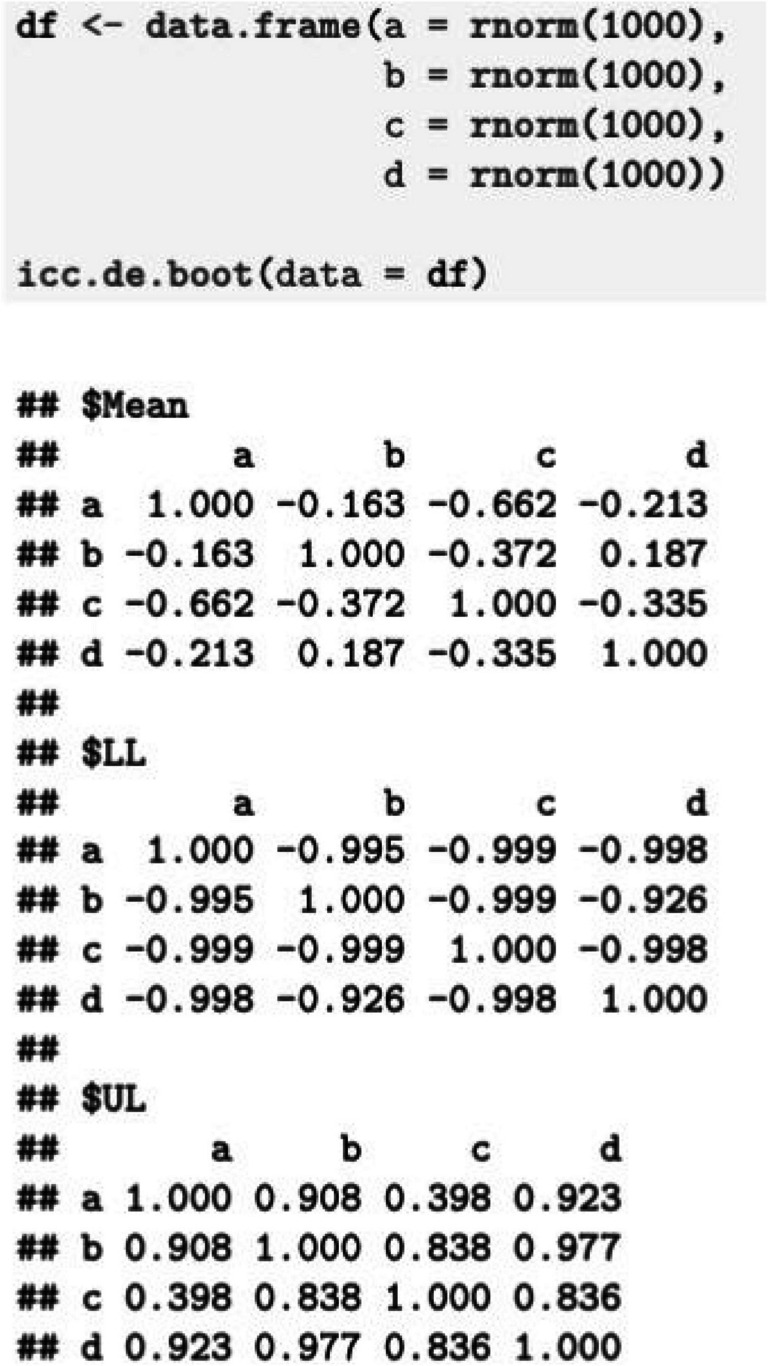
Example for the use of the icc.de.boot function. *Note*. Mean indicates the point estimate of the ICC_DE_, and LL and UL denote the lower and upper boundaries of the confidence interval, respectively

To provide a pertinent significance testing approach, the *icc.de.boot* function from the R package *iccde* (version 0.3.6; Blötner & Grosz, 2024) computes bootstrap confidence intervals for ICC_DE_s, given the desired α-level. The only mandatory input of the function reflects the argument data. It requires entering a data frame of variables (columns) and participants (rows) containing the test scores of ostensibly jingled/jangled constructs as well as test scores of the constructs with which correlations should be tested. Figure 3 provides the output for simulated data with four variables from 1,000 participants. By default, the function computes 95% confidence intervals from 1,000 bootstrap samples, but the confidence level and the number of bootstrap samples can be edited.

If the bootstrap confidence interval of the empirically observed ICC_DE_ exceeds the threshold derived using the above formula, findings militate in favor of a strong jangle fallacy (i.e., nomological networks overlap significantly more strongly than expected, suggesting that the measures assess the same construct) or against a strong jingle fallacy (nomological networks exhibit sufficient overlaps to conclude that the measures assess the same construct).

It is important to note that the above proposal for the derivation of a cutoff for the similarity between nomological networks builds on conceptual considerations, but it does not consider measurement errors. Thus, it is important to clarify how measurement error impacts the empirically observed ICC_DE_ when individual correlations of potentially jingled/jangled test scores with validation criteria are attenuated due to unreliability. To acknowledge this, one might calculate ICC_DE_s from latent factor correlations from confirmatory factor analysis or apply attenuation correction to manifest correlations. Due to easier implementation and lower computational requirements, however, most scholars who evaluate jingle or jangle fallacies presumably examine correlations between manifest test scores (hereafter raw correlations) rather than disattenuated correlations or correlations between latent factors.

**Fig. 4:**
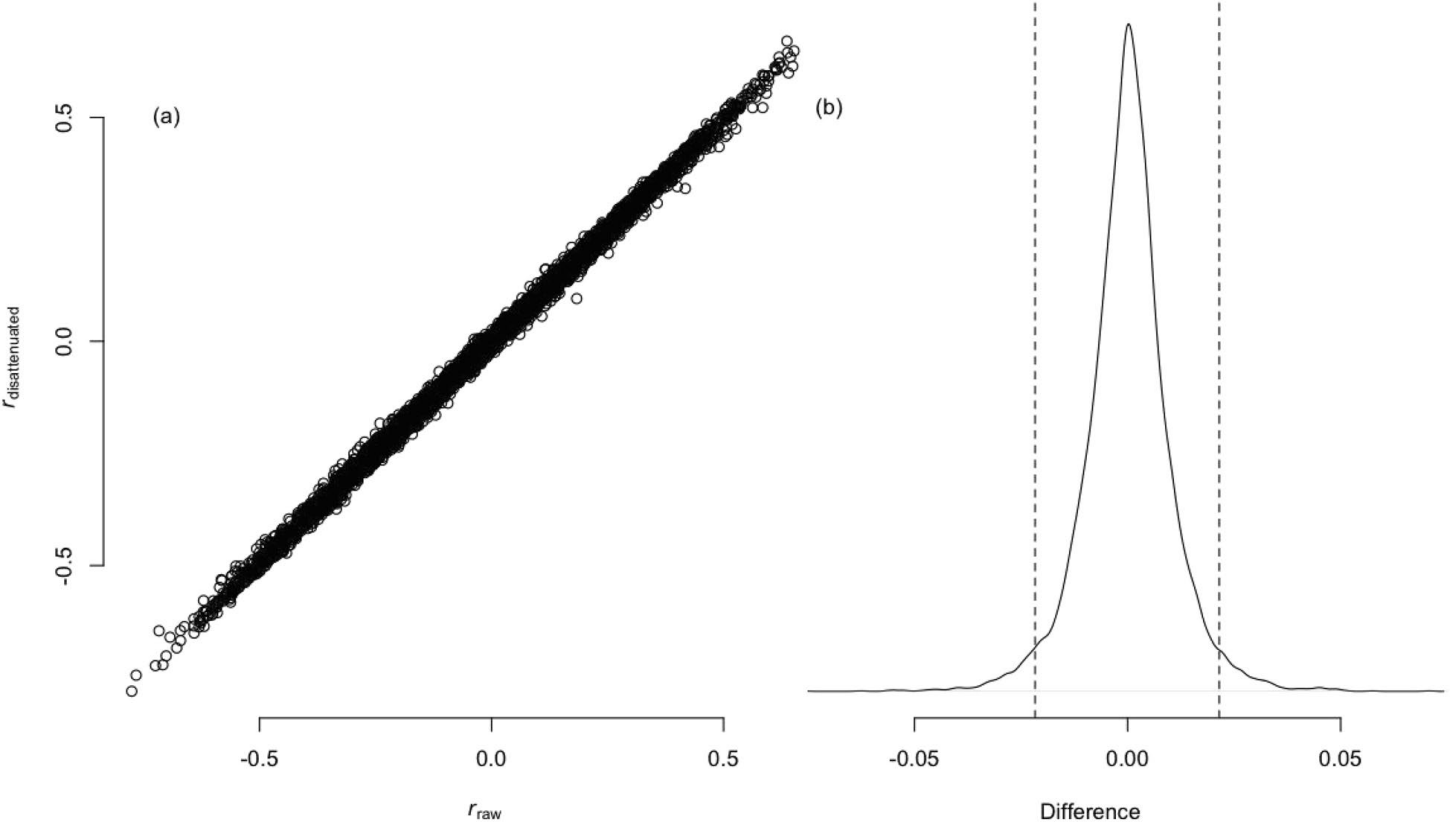
Relations and differences between ICC_DE_s derived from raw and disattenuated correlations. *Note*. **a** Scatterplot for the relationship between ICC_DE_s derived from raw versus disattenuated correlations. **b** Distribution of the difference between the ICC_DE_s derived from raw versus disattenuated correlations

To evaluate the extent to which an ICC_DE_ that rests on raw correlations differs from the corresponding ICC_DE_ that rests on disattenuated correlations, I conducted a simulation study (see OSF supplement https://osf.io/4um8a). I simulated 10,000 pairs of vectors of 20 raw correlation coefficients each (ranging from *r* = −.60 to *r* = .60) and computed the ICC_DE_ for each of the 10,000 pairs.^1^ Furthermore, I computed the corresponding ICC_DE_ for each pair of vectors of disattenuated correlation coefficients (i.e., vectors of correlations that are corrected for measurement error). Thereby, I simulated reliability levels ranging from *r*_xx_ = .70 to *r*_xx_ = 1.00. As can be seen in Fig. 4, differences between ICC_DE_s derived from raw correlations vis-à-vis disattenuated correlations were negligible because the ICC_DE_s computed from raw versus disattenuated correlations were themselves correlated at *r* = .999, and the 95% confidence interval of the differences ranged from −.022 to .022 (see the dashed vertical lines in panel c in Fig. 4). Thus, computing ICC_DE_s with disattenuated correlations does not elicit particular advantages over analyses of raw correlations.

## Conclusion

Arguably, deriving unique and shared elements with the elemental approach is very subjective, as most psychological theories do not provide sufficient formalization to obtain markedly high inter-rater agreement of these elements. Likewise, the devised formula to derive context-specific thresholds for critical ICC_DE_s is quite simple. Yet, given the incorporation of specific, content-related considerations and ideas that are tailored to the research question at hand, it stands to reason that the outlined formula is more appropriate than abstract and vague cutoffs or rules of thumb derived from gut feelings that are supposed to hold for all imaginable tests of jingle/jangle fallacies. Of note, extremely low as well as extremely high cutoffs (e.g., ICC_DE, crit_ = .20 and .95) as derived from the above formula that are very easy or very hard to exceed should raise doubts about the eligibility of the concretely employed set of validation criteria and render the set of criteria uninformative to answer the research question under scrutiny. That said, I would like to encourage future research to employ, challenge, and (if necessary) refine the applicability of the formula.

## Data Availability

Data sharing is not applicable because no real data were collected or analyzed during the current study.

## Footnotes

1 The decisions about the selected number of correlations per vector and the range between −.60 and .60 were arbitrary. However, similar conclusions arise for other numbers of correlations per vector (50, 100) and other ranges of correlations (−.70 ≤ *r*s ≤ .70; see OSF supplement). Note that the upper and lower boundaries for the correlations must not be too high, because otherwise, disattenuated correlations could exceed ±1.

## Code availability

All *R* codes used for the present manuscript are available at https://osf.io/v5dte/.
